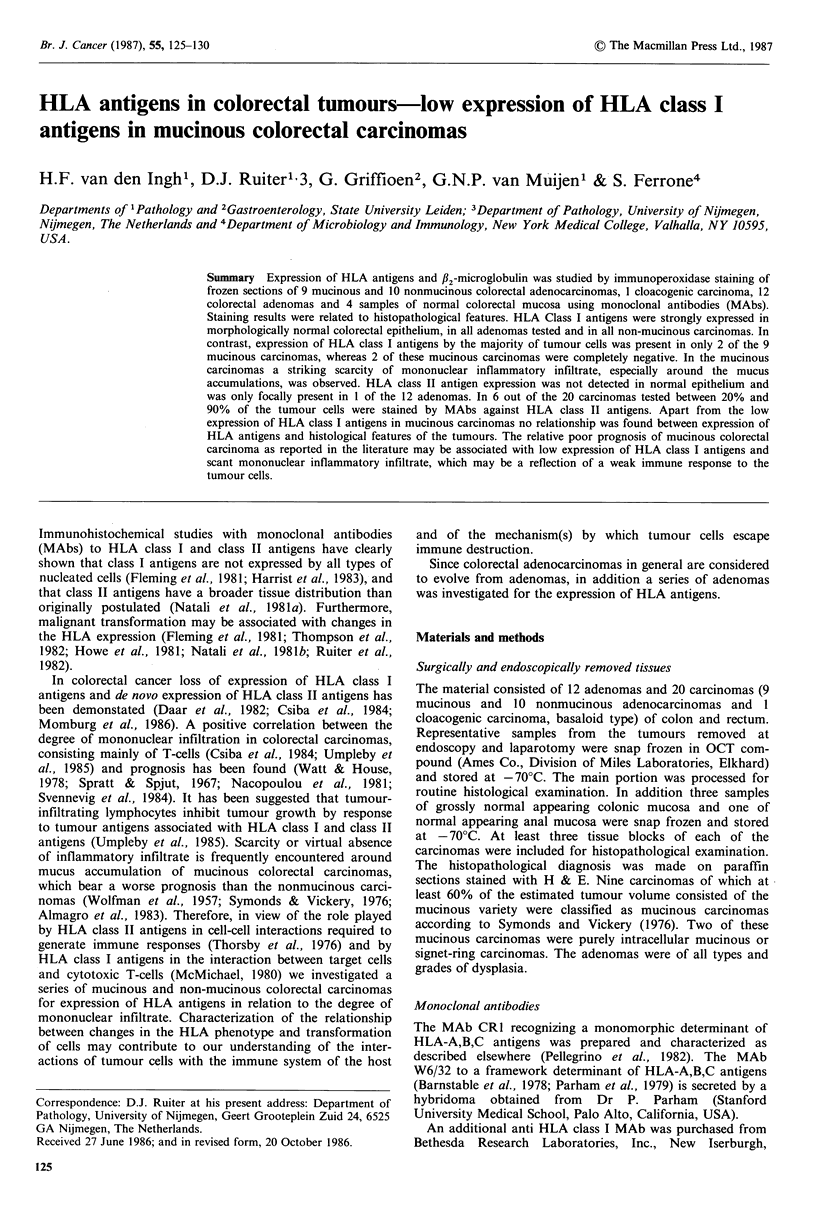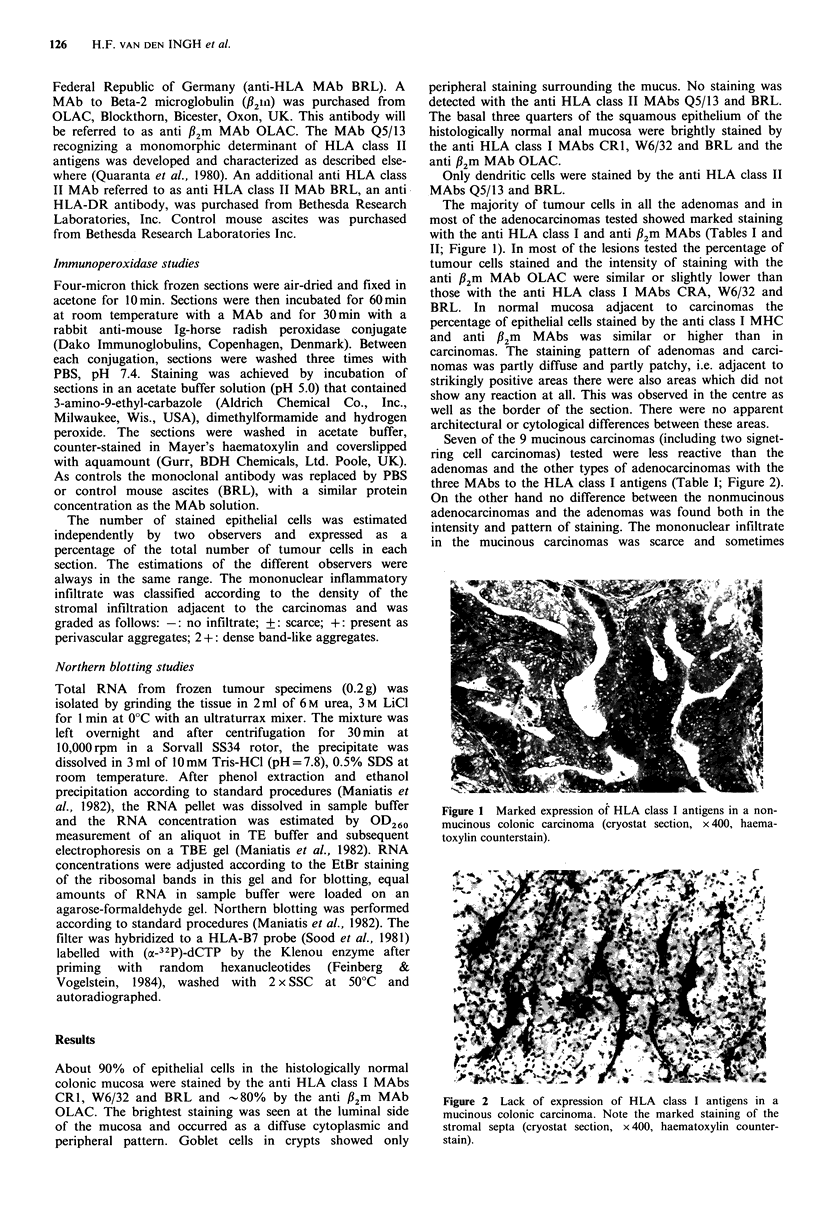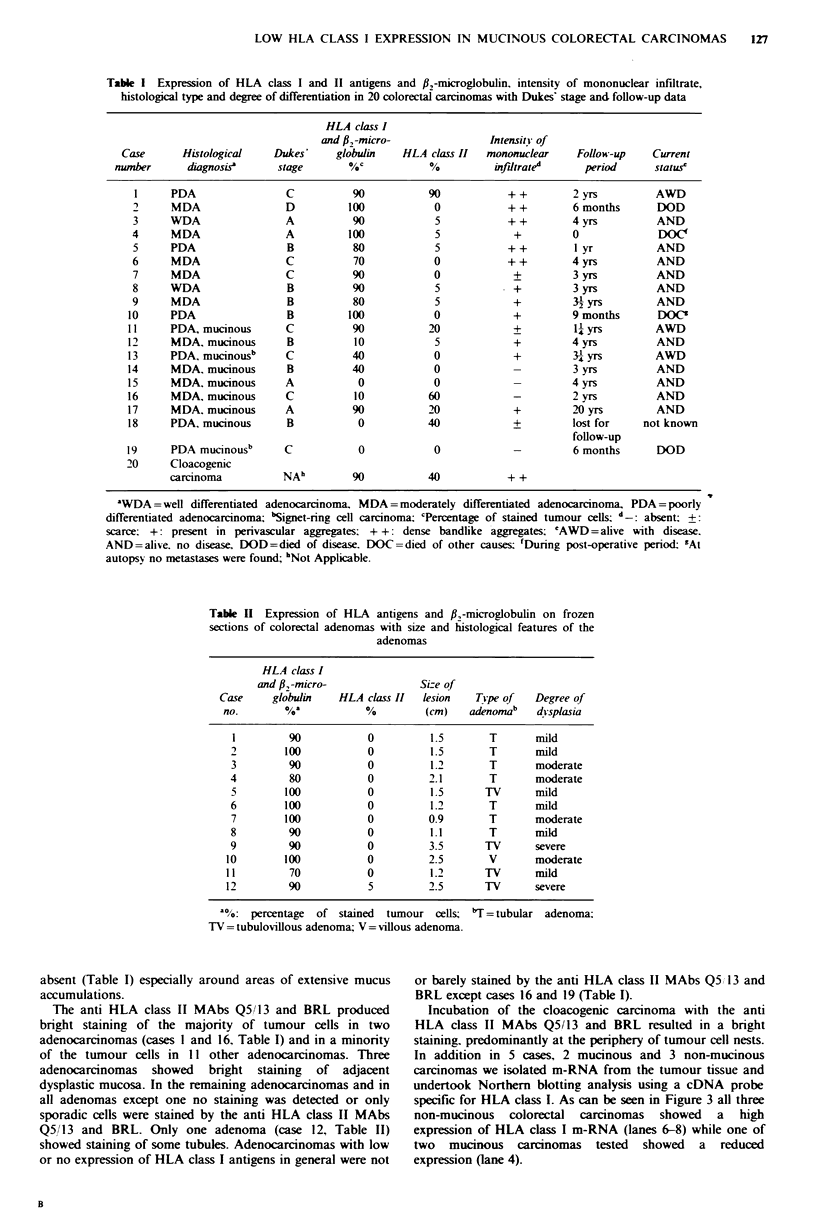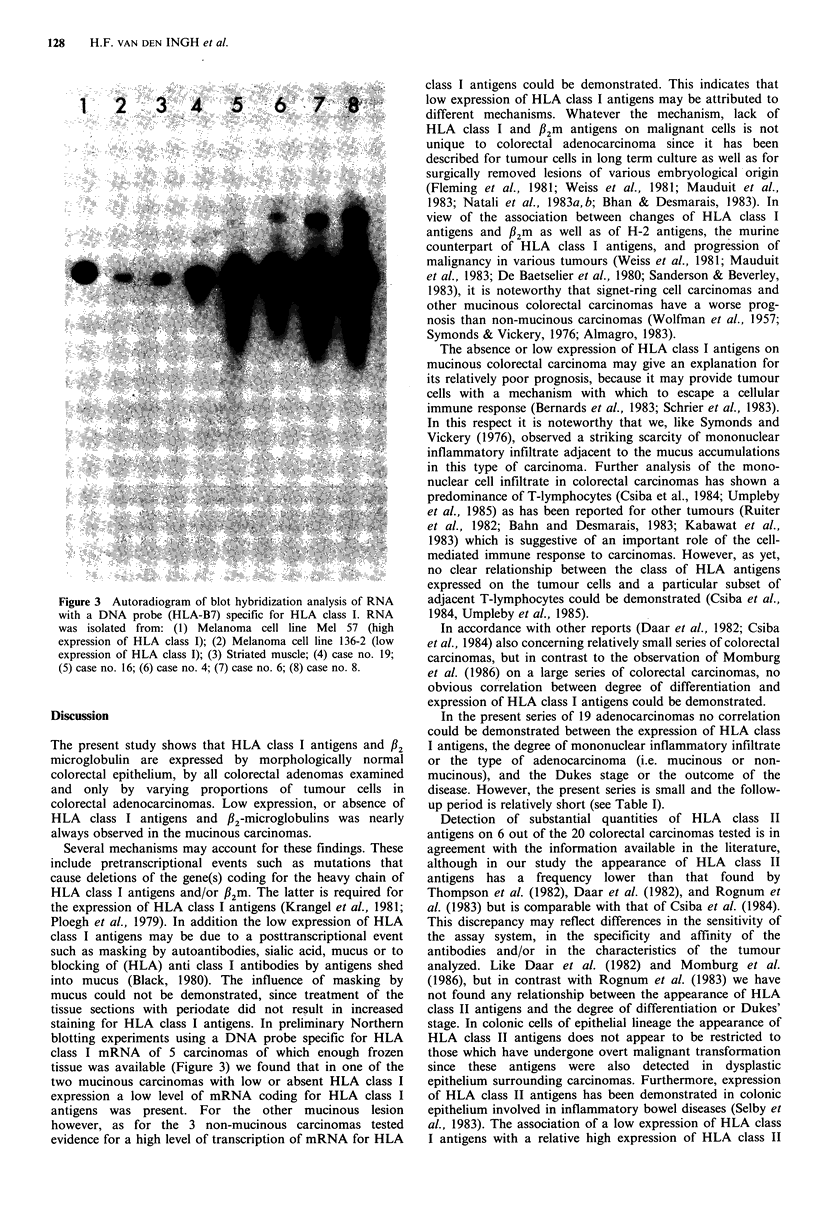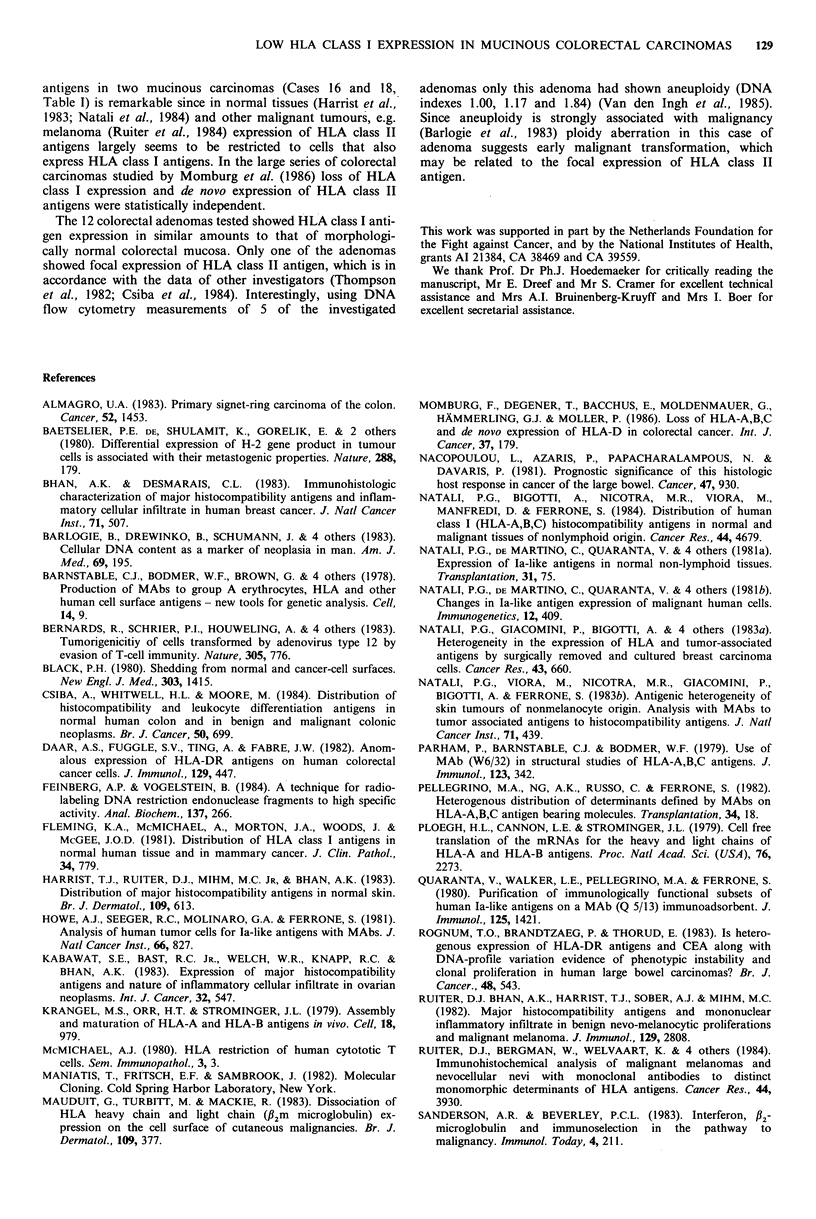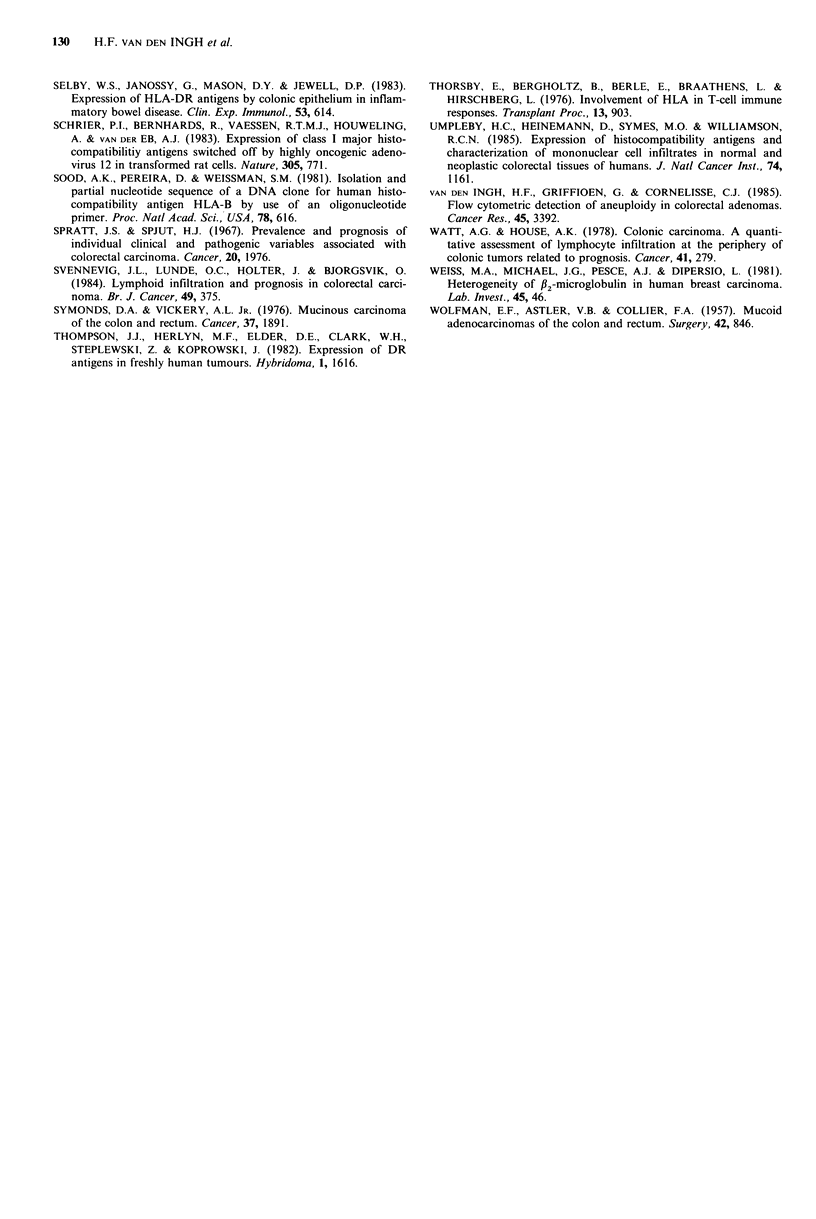# HLA antigens in colorectal tumours--low expression of HLA class I antigens in mucinous colorectal carcinomas.

**DOI:** 10.1038/bjc.1987.26

**Published:** 1987-02

**Authors:** H. F. van den Ingh, D. J. Ruiter, G. Griffioen, G. N. van Muijen, S. Ferrone

## Abstract

**Images:**


*Br. J. Cancer* (1987), **55**, 125–130 © The Macmillan Press Ltd., 1987

### HLA antigens in colorectal tumours—low expression of HLA class I antigens in mucinous colorectal carcinomas

H.F. van den Ingh[1], D.J. Ruiter[1,3], G. Griffioen[2], G.N.P. van Muijen[1] & S. Ferrone[4]

*Departments of [1]Pathology and [2]Gastroenterology, State University Leiden; [3]Department of Pathology, University of Nijmegen, Nijmegen, The Netherlands and [4]Department of Microbiology and Immunology, New York Medical College, Valhalla, NY 10595, USA.*

**Summary** Expression of HLA antigens and $\beta_2$-microglobulin was studied by immunoperoxidase staining of frozen sections of 9 mucinous and 10 nonmucinous colorectal adenocarcinomas, 1 cloacogenic carcinoma, 12 colorectal adenomas and 4 samples of normal colorectal mucosa using monoclonal antibodies (MAbs). Staining results were related to histopathological features. HLA Class I antigens were strongly expressed in morphologically normal colorectal epithelium, in all adenomas tested and in all non-mucinous carcinomas. In contrast, expression of HLA class I antigens by the majority of tumour cells was present in only 2 of the 9 mucinous carcinomas, whereas 2 of these mucinous carcinomas were completely negative. In the mucinous carcinomas a striking scarcity of mononuclear inflammatory infiltrate, especially around the mucus accumulations, was observed. HLA class II antigen expression was not detected in normal epithelium and was only focally present in 1 of the 12 adenomas. In 6 out of the 20 carcinomas tested between 20% and 90% of the tumour cells were stained by MAbs against HLA class II antigens. Apart from the low expression of HLA class I antigens in mucinous carcinomas no relationship was found between expression of HLA antigens and histological features of the tumours. The relative poor prognosis of mucinous colorectal carcinoma as reported in the literature may be associated with low expression of HLA class I antigens and scant mononuclear inflammatory infiltrate, which may be a reflection of a weak immune response to the tumour cells.

Immunohistochemical studies with monoclonal antibodies (MAbs) to HLA class I and class II antigens have clearly shown that class I antigens are not expressed by all types of nucleated cells (Fleming *et al.*, 1981; Harrist *et al.*, 1983), and that class II antigens have a broader tissue distribution than originally postulated (Natali *et al.*, 1981*a*). Furthermore, malignant transformation may be associated with changes in the HLA expression (Fleming *et al.*, 1981; Thompson *et al.*, 1982; Howe *et al.*, 1981; Natali *et al.*, 1981*b*; Ruiter *et al.*, 1982).

In colorectal cancer loss of expression of HLA class I antigens and *de novo* expression of HLA class II antigens has been demonstated (Daar *et al.*, 1982; Csiba *et al.*, 1984; Momburg *et al.*, 1986). A positive correlation between the degree of mononuclear infiltration in colorectal carcinomas, consisting mainly of T-cells (Csiba *et al.*, 1984; Umpleby *et al.*, 1985) and prognosis has been found (Watt & House, 1978; Spratt & Spjut, 1967; Nacopoulou *et al.*, 1981; Svennevig *et al.*, 1984). It has been suggested that tumour-infiltrating lymphocytes inhibit tumour growth by response to tumour antigens associated with HLA class I and class II antigens (Umpleby *et al.*, 1985). Scarcity or virtual absence of inflammatory infiltrate is frequently encountered around mucus accumulation of mucinous colorectal carcinomas, which bear a worse prognosis than the nonmucinous carcinomas (Wolfman *et al.*, 1957; Symonds & Vickery, 1976; Almagro *et al.*, 1983). Therefore, in view of the role played by HLA class II antigens in cell-cell interactions required to generate immune responses (Thorsby *et al.*, 1976) and by HLA class I antigens in the interaction between target cells and cytotoxic T-cells (McMichael, 1980) we investigated a series of mucinous and non-mucinous colorectal carcinomas for expression of HLA antigens in relation to the degree of mononuclear infiltrate. Characterization of the relationship between changes in the HLA phenotype and transformation of cells may contribute to our understanding of the interactions of tumour cells with the immune system of the host and of the mechanism(s) by which tumour cells escape immune destruction.

Since colorectal adenocarcinomas in general are considered to evolve from adenomas, in addition a series of adenomas was investigated for the expression of HLA antigens.

### Materials and methods

### *Surgically and endoscopically removed tissues*

The material consisted of 12 adenomas and 20 carcinomas (9 mucinous and 10 nonmucinous adenocarcinomas and 1 cloacogenic carcinoma, basaloid type) of colon and rectum. Representative samples from the tumours removed at endoscopy and laparotomy were snap frozen in OCT compound (Ames Co., Division of Miles Laboratories, Elkhard) and stored at −70°C. The main portion was processed for routine histological examination. In addition three samples of grossly normal appearing colonic mucosa and one of normal appearing anal mucosa were snap frozen and stored at −70°C. At least three tissue blocks of each of the carcinomas were included for histopathological examination. The histopathological diagnosis was made on paraffin sections stained with H & E. Nine carcinomas of which at least 60% of the estimated tumour volume consisted of the mucinous variety were classified as mucinous carcinomas according to Symonds and Vickery (1976). Two of these mucinous carcinomas were purely intracellular mucinous or signet-ring carcinomas. The adenomas were of all types and grades of dysplasia.

### *Monoclonal antibodies*

The MAb CR1 recognizing a monomorphic determinant of HLA-A,B,C antigens was prepared and characterized as described elsewhere (Pellegrino *et al.*, 1982). The MAb W6/32 to a framework determinant of HLA-A,B,C antigens (Barnstable *et al.*, 1978; Parham *et al.*, 1979) is secreted by a hybridoma obtained from Dr P. Parham (Stanford University Medical School, Palo Alto, California, USA).

An additional anti HLA class I MAb was purchased from Bethesda Research Laboratories, Inc., New Iserburgh,

Correspondence: D.J. Ruiter at his present address: Department of Pathology, University of Nijmegen, Geert Grooteplein Zuid 24, 6525 GA Nijmegen, The Netherlands.
Received 27 June 1986; and in revised form, 20 October 1986.

Federal Republic of Germany (anti-HLA MAb BRL). A MAb to Beta-2 microglobulin ($\beta_2$m) was purchased from OLAC, Blockthorn, Bicester, Oxon, UK. This antibody will be referred to as anti $\beta_2$m MAb OLAC. The MAb Q5/13 recognizing a monomorphic determinant of HLA class II antigens was developed and characterized as described elsewhere (Quaranta *et al.*, 1980). An additional anti HLA class II MAb referred to as anti HLA class II MAb BRL, an anti HLA-DR antibody, was purchased from Bethesda Research Laboratories, Inc. Control mouse ascites was purchased from Bethesda Research Laboratories Inc.

*Immunoperoxidase studies*

Four-micron thick frozen sections were air-dried and fixed in acetone for 10 min. Sections were then incubated for 60 min at room temperature with a MAb and for 30 min with a rabbit anti-mouse Ig-horse radish peroxidase conjugate (Dako Immunoglobulins, Copenhagen, Denmark). Between each conjugation, sections were washed three times with PBS, pH 7.4. Staining was achieved by incubation of sections in an acetate buffer solution (pH 5.0) that contained 3-amino-9-ethyl-carbazole (Aldrich Chemical Co., Inc., Milwaukee, Wis., USA), dimethylformamide and hydrogen peroxide. The sections were washed in acetate buffer, counter-stained in Mayer's haematoxylin and coverslipped with aquamount (Gurr, BDH Chemicals, Ltd. Poole, UK). As controls the monoclonal antibody was replaced by PBS or control mouse ascites (BRL), with a similar protein concentration as the MAb solution.

The number of stained epithelial cells was estimated independently by two observers and expressed as a percentage of the total number of tumour cells in each section. The estimations of the different observers were always in the same range. The mononuclear inflammatory infiltrate was classified according to the density of the stromal infiltration adjacent to the carcinomas and was graded as follows: −: no infiltrate; ±: scarce; +: present as perivascular aggregates; 2+: dense band-like aggregates.

*Northern blotting studies*

Total RNA from frozen tumour specimens (0.2 g) was isolated by grinding the tissue in 2 ml of 6 M urea, 3 M LiCl for 1 min at 0°C with an ultraturrax mixer. The mixture was left overnight and after centrifugation for 30 min at 10,000 rpm in a Sorvall SS34 rotor, the precipitate was dissolved in 3 ml of 10 mM Tris-HCl (pH = 7.8), 0.5% SDS at room temperature. After phenol extraction and ethanol precipitation according to standard procedures (Maniatis *et al.*, 1982), the RNA pellet was dissolved in sample buffer and the RNA concentration was estimated by $OD_{260}$ measurement of an aliquot in TE buffer and subsequent electrophoresis on a TBE gel (Maniatis *et al.*, 1982). RNA concentrations were adjusted according to the EtBr staining of the ribosomal bands in this gel and for blotting, equal amounts of RNA in sample buffer were loaded on an agarose-formaldehyde gel. Northern blotting was performed according to standard procedures (Maniatis *et al.*, 1982). The filter was hybridized to a HLA-B7 probe (Sood *et al.*, 1981) labelled with ($\alpha$-$^{32}$P)-dCTP by the Klenou enzyme after priming with random hexanucleotides (Feinberg & Vogelstein, 1984), washed with 2 × SSC at 50°C and autoradiographed.

### Results

About 90% of epithelial cells in the histologically normal colonic mucosa were stained by the anti HLA class I MAbs CR1, W6/32 and BRL and ~80% by the anti $\beta_2$m MAb OLAC. The brightest staining was seen at the luminal side of the mucosa and occurred as a diffuse cytoplasmic and peripheral pattern. Goblet cells in crypts showed only peripheral staining surrounding the mucus. No staining was detected with the anti HLA class II MAbs Q5/13 and BRL. The basal three quarters of the squamous epithelium of the histologically normal anal mucosa were brightly stained by the anti HLA class I MAbs CR1, W6/32 and BRL and the anti $\beta_2$m MAb OLAC.

Only dendritic cells were stained by the anti HLA class II MAbs Q5/13 and BRL.

The majority of tumour cells in all the adenomas and in most of the adenocarcinomas tested showed marked staining with the anti HLA class I and anti $\beta_2$m MAbs (Tables I and II; Figure 1). In most of the lesions tested the percentage of tumour cells stained and the intensity of staining with the anti $\beta_2$m MAb OLAC were similar or slightly lower than those with the anti HLA class I MAbs CRA, W6/32 and BRL. In normal mucosa adjacent to carcinomas the percentage of epithelial cells stained by the anti class I MHC and anti $\beta_2$m MAbs was similar or higher than in carcinomas. The staining pattern of adenomas and carcinomas was partly diffuse and partly patchy, i.e. adjacent to strikingly positive areas there were also areas which did not show any reaction at all. This was observed in the centre as well as the border of the section. There were no apparent architectural or cytological differences between these areas.

Seven of the 9 mucinous carcinomas (including two signet-ring cell carcinomas) tested were less reactive than the adenomas and the other types of adenocarcinomas with the three MAbs to the HLA class I antigens (Table I; Figure 2). On the other hand no difference between the nonmucinous adenocarcinomas and the adenomas was found both in the intensity and pattern of staining. The mononuclear infiltrate in the mucinous carcinomas was scarce and sometimes

**Figure 1** Marked expression of HLA class I antigens in a non-mucinous colonic carcinoma (cryostat section, ×400, haematoxylin counterstain).

**Figure 2** Lack of expression of HLA class I antigens in a mucinous colonic carcinoma. Note the marked staining of the stromal septa (cryostat section, ×400, haematoxylin counterstain).

**Table I** Expression of HLA class I and II antigens and $\beta_2$-microglobulin, intensity of mononuclear infiltrate, histological type and degree of differentiation in 20 colorectal carcinomas with Dukes' stage and follow-up data

| *Case number* | *Histological diagnosis*[a] | *Dukes' stage* | *HLA class I and $\beta_2$-microglobulin %*[c] | *HLA class II %* | *Intensity of mononuclear infiltrate*[d] | *Follow-up period* | *Current status*[e] |
|---|---|---|---|---|---|---|---|
| 1 | PDA | C | 90 | 90 | + + | 2 yrs | AWD |
| 2 | MDA | D | 100 | 0 | + + | 6 months | DOD |
| 3 | WDA | A | 90 | 5 | + + | 4 yrs | AND |
| 4 | MDA | A | 100 | 5 | + | 0 | DOC[f] |
| 5 | PDA | B | 80 | 5 | + + | 1 yr | AND |
| 6 | MDA | C | 70 | 0 | + + | 4 yrs | AND |
| 7 | MDA | C | 90 | 0 | ± | 3 yrs | AND |
| 8 | WDA | B | 90 | 5 | + | 3 yrs | AND |
| 9 | MDA | B | 80 | 5 | + | 3½ yrs | AND |
| 10 | PDA | B | 100 | 0 | + | 9 months | DOC[g] |
| 11 | PDA, mucinous | C | 90 | 20 | ± | 1¼ yrs | AWD |
| 12 | MDA, mucinous | B | 10 | 5 | + | 4 yrs | AND |
| 13 | PDA, mucinous[b] | C | 40 | 0 | + | 3¼ yrs | AWD |
| 14 | MDA, mucinous | B | 40 | 0 | – | 3 yrs | AND |
| 15 | MDA, mucinous | A | 0 | 0 | – | 4 yrs | AND |
| 16 | MDA, mucinous | C | 10 | 60 | – | 2 yrs | AND |
| 17 | MDA, mucinous | A | 90 | 20 | + | 20 yrs | AND |
| 18 | PDA, mucinous | B | 0 | 40 | ± | lost for follow-up | not known |
| 19 | PDA mucinous[b] | C | 0 | 0 | – | 6 months | DOD |
| 20 | Cloacogenic carcinoma | NA[h] | 90 | 40 | + + | | |

[a]WDA = well differentiated adenocarcinoma, MDA = moderately differentiated adenocarcinoma, PDA = poorly differentiated adenocarcinoma; [b]Signet-ring cell carcinoma; [c]Percentage of stained tumour cells; [d]–: absent; ±: scarce; +: present in perivascular aggregates; + +: dense bandlike aggregates; [e]AWD = alive with disease, AND = alive, no disease, DOD = died of disease, DOC = died of other causes; [f]During post-operative period; [g]At autopsy no metastases were found; [h]Not Applicable.

**Table II** Expression of HLA antigens and $\beta_2$-microglobulin on frozen sections of colorectal adenomas with size and histological features of the adenomas

| *Case no.* | *HLA class I and $\beta_2$-microglobulin %*[a] | *HLA class II %* | *Size of lesion (cm)* | *Type of adenoma*[b] | *Degree of dysplasia* |
|---|---|---|---|---|---|
| 1 | 90 | 0 | 1.5 | T | mild |
| 2 | 100 | 0 | 1.5 | T | mild |
| 3 | 90 | 0 | 1.2 | T | moderate |
| 4 | 80 | 0 | 2.1 | T | moderate |
| 5 | 100 | 0 | 1.5 | TV | mild |
| 6 | 100 | 0 | 1.2 | T | mild |
| 7 | 100 | 0 | 0.9 | T | moderate |
| 8 | 90 | 0 | 1.1 | T | mild |
| 9 | 90 | 0 | 3.5 | TV | severe |
| 10 | 100 | 0 | 2.5 | V | moderate |
| 11 | 70 | 0 | 1.2 | TV | mild |
| 12 | 90 | 5 | 2.5 | TV | severe |

[a]%: percentage of stained tumour cells; [b]T = tubular adenoma; TV = tubulovillous adenoma; V = villous adenoma.

absent (Table I) especially around areas of extensive mucus accumulations.

The anti HLA class II MAbs Q5/13 and BRL produced bright staining of the majority of tumour cells in two adenocarcinomas (cases 1 and 16, Table I) and in a minority of the tumour cells in 11 other adenocarcinomas. Three adenocarcinomas showed bright staining of adjacent dysplastic mucosa. In the remaining adenocarcinomas and in all adenomas except one no staining was detected or only sporadic cells were stained by the anti HLA class II MAbs Q5/13 and BRL. Only one adenoma (case 12, Table II) showed staining of some tubules. Adenocarcinomas with low or no expression of HLA class I antigens in general were not or barely stained by the anti HLA class II MAbs Q5/13 and BRL except cases 16 and 19 (Table I).

Incubation of the cloacogenic carcinoma with the anti HLA class II MAbs Q5/13 and BRL resulted in a bright staining, predominantly at the periphery of tumour cell nests. In addition in 5 cases, 2 mucinous and 3 non-mucinous carcinomas we isolated m-RNA from the tumour tissue and undertook Northern blotting analysis using a cDNA probe specific for HLA class I. As can be seen in Figure 3 all three non-mucinous colorectal carcinomas showed a high expression of HLA class I m-RNA (lanes 6–8) while one of two mucinous carcinomas tested showed a reduced expression (lane 4).

**Figure 3** Autoradiogram of blot hybridization analysis of RNA with a DNA probe (HLA-B7) specific for HLA class I. RNA was isolated from: (1) Melanoma cell line Mel 57 (high expression of HLA class I); (2) Melanoma cell line 136-2 (low expression of HLA class I); (3) Striated muscle; (4) case no. 19; (5) case no. 16; (6) case no. 4; (7) case no. 6; (8) case no. 8.

### Discussion

The present study shows that HLA class I antigens and $\beta_2$ microglobulin are expressed by morphologically normal colorectal epithelium, by all colorectal adenomas examined and only by varying proportions of tumour cells in colorectal adenocarcinomas. Low expression, or absence of HLA class I antigens and $\beta_2$-microglobulins was nearly always observed in the mucinous carcinomas.

Several mechanisms may account for these findings. These include pretranscriptional events such as mutations that cause deletions of the gene(s) coding for the heavy chain of HLA class I antigens and/or $\beta_2$m. The latter is required for the expression of HLA class I antigens (Krangel *et al.*, 1981; Ploegh *et al.*, 1979). In addition the low expression of HLA class I antigens may be due to a posttranscriptional event such as masking by autoantibodies, sialic acid, mucus or to blocking of (HLA) anti class I antibodies by antigens shed into mucus (Black, 1980). The influence of masking by mucus could not be demonstrated, since treatment of the tissue sections with periodate did not result in increased staining for HLA class I antigens. In preliminary Northern blotting experiments using a DNA probe specific for HLA class I mRNA of 5 carcinomas of which enough frozen tissue was available (Figure 3) we found that in one of the two mucinous carcinomas with low or absent HLA class I expression a low level of mRNA coding for HLA class I antigens was present. For the other mucinous lesion however, as for the 3 non-mucinous carcinomas tested evidence for a high level of transcription of mRNA for HLA class I antigens could be demonstrated. This indicates that low expression of HLA class I antigens may be attributed to different mechanisms. Whatever the mechanism, lack of HLA class I and $\beta_2$m antigens on malignant cells is not unique to colorectal adenocarcinoma since it has been described for tumour cells in long term culture as well as for surgically removed lesions of various embryological origin (Fleming *et al.*, 1981; Weiss *et al.*, 1981; Mauduit *et al.*, 1983; Natali *et al.*, 1983*a,b*; Bhan & Desmarais, 1983). In view of the association between changes of HLA class I antigens and $\beta_2$m as well as of H-2 antigens, the murine counterpart of HLA class I antigens, and progression of malignancy in various tumours (Weiss *et al.*, 1981; Mauduit *et al.*, 1983; De Baetselier *et al.*, 1980; Sanderson & Beverley, 1983), it is noteworthy that signet-ring cell carcinomas and other mucinous colorectal carcinomas have a worse prognosis than non-mucinous carcinomas (Wolfman *et al.*, 1957; Symonds & Vickery, 1976; Almagro, 1983).

The absence or low expression of HLA class I antigens on mucinous colorectal carcinoma may give an explanation for its relatively poor prognosis, because it may provide tumour cells with a mechanism with which to escape a cellular immune response (Bernards *et al.*, 1983; Schrier *et al.*, 1983). In this respect it is noteworthy that we, like Symonds and Vickery (1976), observed a striking scarcity of mononuclear inflammatory infiltrate adjacent to the mucus accumulations in this type of carcinoma. Further analysis of the mononuclear cell infiltrate in colorectal carcinomas has shown a predominance of T-lymphocytes (Csiba et al., 1984; Umpleby *et al.*, 1985) as has been reported for other tumours (Ruiter *et al.*, 1982; Bahn and Desmarais, 1983; Kabawat *et al.*, 1983) which is suggestive of an important role of the cell-mediated immune response to carcinomas. However, as yet, no clear relationship between the class of HLA antigens expressed on the tumour cells and a particular subset of adjacent T-lymphocytes could be demonstrated (Csiba *et al.*, 1984, Umpleby *et al.*, 1985).

In accordance with other reports (Daar *et al.*, 1982; Csiba *et al.*, 1984) also concerning relatively small series of colorectal carcinomas, but in contrast to the observation of Momburg *et al.* (1986) on a large series of colorectal carcinomas, no obvious correlation between degree of differentiation and expression of HLA class I antigens could be demonstrated.

In the present series of 19 adenocarcinomas no correlation could be demonstrated between the expression of HLA class I antigens, the degree of mononuclear inflammatory infiltrate or the type of adenocarcinoma (i.e. mucinous or non-mucinous), and the Dukes stage or the outcome of the disease. However, the present series is small and the follow-up period is relatively short (see Table I).

Detection of substantial quantities of HLA class II antigens on 6 out of the 20 colorectal carcinomas tested is in agreement with the information available in the literature, although in our study the appearance of HLA class II antigens has a frequency lower than that found by Thompson *et al.* (1982), Daar *et al.* (1982), and Rognum *et al.* (1983) but is comparable with that of Csiba *et al.* (1984). This discrepancy may reflect differences in the sensitivity of the assay system, in the specificity and affinity of the antibodies and/or in the characteristics of the tumour analyzed. Like Daar *et al.* (1982) and Momburg *et al.* (1986), but in contrast with Rognum *et al.* (1983) we have not found any relationship between the appearance of HLA class II antigens and the degree of differentiation or Dukes' stage. In colonic cells of epithelial lineage the appearance of HLA class II antigens does not appear to be restricted to those which have undergone overt malignant transformation since these antigens were also detected in dysplastic epithelium surrounding carcinomas. Furthermore, expression of HLA class II antigens has been demonstrated in colonic epithelium involved in inflammatory bowel diseases (Selby *et al.*, 1983). The association of a low expression of HLA class I antigens with a relative high expression of HLA class II

antigens in two mucinous carcinomas (Cases 16 and 18, Table I) is remarkable since in normal tissues (Harrist *et al.*, 1983; Natali *et al.*, 1984) and other malignant tumours, e.g. melanoma (Ruiter *et al.*, 1984) expression of HLA class II antigens largely seems to be restricted to cells that also express HLA class I antigens. In the large series of colorectal carcinomas studied by Momburg *et al.* (1986) loss of HLA class I expression and *de novo* expression of HLA class II antigens were statistically independent.

The 12 colorectal adenomas tested showed HLA class I antigen expression in similar amounts to that of morphologically normal colorectal mucosa. Only one of the adenomas showed focal expression of HLA class II antigen, which is in accordance with the data of other investigators (Thompson *et al.*, 1982; Csiba *et al.*, 1984). Interestingly, using DNA flow cytometry measurements of 5 of the investigated adenomas only this adenoma had shown aneuploidy (DNA indexes 1.00, 1.17 and 1.84) (Van den Ingh *et al.*, 1985). Since aneuploidy is strongly associated with malignancy (Barlogie *et al.*, 1983) ploidy aberration in this case of adenoma suggests early malignant transformation, which may be related to the focal expression of HLA class II antigen.

This work was supported in part by the Netherlands Foundation for the Fight against Cancer, and by the National Institutes of Health, grants AI 21384, CA 38469 and CA 39559.

We thank Prof. Dr Ph.J. Hoedemaeker for critically reading the manuscript, Mr E. Dreef and Mr S. Cramer for excellent technical assistance and Mrs A.I. Bruinenberg-Kruyff and Mrs I. Boer for excellent secretarial assistance.

### References

ALMAGRO, U.A. (1983). Primary signet-ring carcinoma of the colon. *Cancer*, **52**, 1453.

BAETSELIER, P.E. DE, SHULAMIT, K., GORELIK, E. & 2 others (1980). Differential expression of H-2 gene product in tumour cells is associated with their metastogenic properties. *Nature*, **288**, 179.

BHAN, A.K. & DESMARAIS, C.L. (1983). Immunohistologic characterization of major histocompatibility antigens and inflammatory cellular infiltrate in human breast cancer. *J. Natl Cancer Inst.*, **71**, 507.

BARLOGIE, B., DREWINKO, B., SCHUMANN, J. & 4 others (1983). Cellular DNA content as a marker of neoplasia in man. *Am. J. Med.*, **69**, 195.

BARNSTABLE, C.J., BODMER, W.F., BROWN, G. & 4 others (1978). Production of MAbs to group A erythrocytes, HLA and other human cell surface antigens – new tools for genetic analysis. *Cell*, **14**, 9.

BERNARDS, R., SCHRIER, P.I., HOUWELING, A. & 4 others (1983). Tumorigenicitiy of cells transformed by adenovirus type 12 by evasion of T-cell immunity. *Nature*, **305**, 776.

BLACK, P.H. (1980). Shedding from normal and cancer-cell surfaces. *New Engl. J. Med.*, **303**, 1415.

CSIBA, A., WHITWELL, H.L. & MOORE, M. (1984). Distribution of histocompatibility and leukocyte differentiation antigens in normal human colon and in benign and malignant colonic neoplasms. *Br. J. Cancer*, **50**, 699.

DAAR, A.S., FUGGLE, S.V., TING, A. & FABRE, J.W. (1982). Anomalous expression of HLA-DR antigens on human colorectal cancer cells. *J. Immunol.*, **129**, 447.

FEINBERG, A.P. & VOGELSTEIN, B. (1984). A technique for radiolabeling DNA restriction endonuclease fragments to high specific activity. *Anal. Biochem.*, **137**, 266.

FLEMING, K.A., MCMICHAEL, A., MORTON, J.A., WOODS, J. & MCGEE, J.O.D. (1981). Distribution of HLA class I antigens in normal human tissue and in mammary cancer. *J. Clin. Pathol.*, **34**, 779.

HARRIST, T.J., RUITER, D.J., MIHM, M.C. JR, & BHAN, A.K. (1983). Distribution of major histocompatibility antigens in normal skin. *Br. J. Dermatol.*, **109**, 613.

HOWE, A.J., SEEGER, R.C., MOLINARO, G.A. & FERRONE, S. (1981). Analysis of human tumor cells for Ia-like antigens with MAbs. *J. Natl Cancer Inst.*, **66**, 827.

KABAWAT, S.E., BAST, R.C. JR., WELCH, W.R., KNAPP, R.C. & BHAN, A.K. (1983). Expression of major histocompatibility antigens and nature of inflammatory cellular infiltrate in ovarian neoplasms. *Int. J. Cancer*, **32**, 547.

KRANGEL, M.S., ORR, H.T. & STROMINGER, J.L. (1979). Assembly and maturation of HLA-A and HLA-B antigens *in vivo*. *Cell*, **18**, 979.

MCMICHAEL, A.J. (1980). HLA restriction of human cytotoxic T cells. *Sem. Immunopathol.*, **3**, 3.

MANIATIS, T., FRITSCH, E.F. & SAMBROOK, J. (1982). Molecular Cloning. Cold Spring Harbor Laboratory, New York.

MAUDUIT, G., TURBITT, M. & MACKIE, R. (1983). Dissociation of HLA heavy chain and light chain ($\beta_2$m microglobulin) expression on the cell surface of cutaneous malignancies. *Br. J. Dermatol.*, **109**, 377.

MOMBURG, F., DEGENER, T., BACCHUS, E., MOLDENMAUER, G., HÄMMERLING, G.J. & MOLLER, P. (1986). Loss of HLA-A,B,C and *de novo* expression of HLA-D in colorectal cancer. *Int. J. Cancer*, **37**, 179.

NACOPOULOU, L., AZARIS, P., PAPACHARALAMPOUS, N. & DAVARIS, P. (1981). Prognostic significance of this histologic host response in cancer of the large bowel. *Cancer*, **47**, 930.

NATALI, P.G., BIGOTTI, A., NICOTRA, M.R., VIORA, M., MANFREDI, D. & FERRONE, S. (1984). Distribution of human class I (HLA-A,B,C) histocompatibility antigens in normal and malignant tissues of nonlymphoid origin. *Cancer Res.*, **44**, 4679.

NATALI, P.G., DE MARTINO, C., QUARANTA, V. & 4 others (1981a). Expression of Ia-like antigens in normal non-lymphoid tissues. *Transplantation*, **31**, 75.

NATALI, P.G., DE MARTINO, C., QUARANTA, V. & 4 others (1981*b*). Changes in Ia-like antigen expression of malignant human cells. *Immunogenetics*, **12**, 409.

NATALI, P.G., GIACOMINI, P., BIGOTTI, A. & 4 others (1983*a*). Heterogeneity in the expression of HLA and tumor-associated antigens by surgically removed and cultured breast carcinoma cells. *Cancer Res.*, **43**, 660.

NATALI, P.G., VIORA, M., NICOTRA, M.R., GIACOMINI, P., BIGOTTI, A. & FERRONE, S. (1983*b*). Antigenic heterogeneity of skin tumours of nonmelanocyte origin. Analysis with MAbs to tumor associated antigens to histocompatibility antigens. *J. Natl Cancer Inst.*, **71**, 439.

PARHAM, P., BARNSTABLE, C.J. & BODMER, W.F. (1979). Use of MAb (W6/32) in structural studies of HLA-A,B,C antigens. *J. Immunol.*, **123**, 342.

PELLEGRINO, M.A., NG, A.K., RUSSO, C. & FERRONE, S. (1982). Heterogenous distribution of determinants defined by MAbs on HLA-A,B,C antigen bearing molecules. *Transplantation*, **34**, 18.

PLOEGH, H.L., CANNON, L.E. & STROMINGER, J.L. (1979). Cell free translation of the mRNAs for the heavy and light chains of HLA-A and HLA-B antigens. *Proc. Natl Acad. Sci.* (*USA*), **76**, 2273.

QUARANTA, V., WALKER, L.E., PELLEGRINO, M.A. & FERRONE, S. (1980). Purification of immunologically functional subsets of human Ia-like antigens on a MAb (Q 5/13) immunoadsorbent. *J. Immunol.*, **125**, 1421.

ROGNUM, T.O., BRANDTZAEG, P. & THORUD, E. (1983). Is heterogenous expression of HLA-DR antigens and CEA along with DNA-profile variation evidence of phenotypic instability and clonal proliferation in human large bowel carcinomas? *Br. J. Cancer.*, **48**, 543.

RUITER, D.J. BHAN, A.K., HARRIST, T.J., SOBER, A.J. & MIHM, M.C. (1982). Major histocompatibility antigens and mononuclear inflammatory infiltrate in benign nevo-melanocytic proliferations and malignant melanoma. *J. Immunol.*, **129**, 2808.

RUITER, D.J., BERGMAN, W., WELVAART, K. & 4 others (1984). Immunohistochemical analysis of malignant melanomas and nevocellular nevi with monoclonal antibodies to distinct monomorphic determinants of HLA antigens. *Cancer Res.*, **44**, 3930.

SANDERSON, A.R. & BEVERLEY, P.C.L. (1983). Interferon, $\beta_2$-microglobulin and immunoselection in the pathway to malignancy. *Immunol. Today*, **4**, 211.

SELBY, W.S., JANOSSY, G., MASON, D.Y. & JEWELL, D.P. (1983). Expression of HLA-DR antigens by colonic epithelium in inflammatory bowel disease. *Clin. Exp. Immunol.*, **53**, 614.

SCHRIER, P.I., BERNHARDS, R., VAESSEN, R.T.M.J., HOUWELING, A. & VAN DER EB, A.J. (1983). Expression of class I major histocompatibilitiy antigens switched off by highly oncogenic adenovirus 12 in transformed rat cells. *Nature*, **305**, 771.

SOOD, A.K., PEREIRA, D. & WEISSMAN, S.M. (1981). Isolation and partial nucleotide sequence of a DNA clone for human histocompatibility antigen HLA-B by use of an oligonucleotide primer. *Proc. Natl Acad. Sci., USA*, **78**, 616.

SPRATT, J.S. & SPJUT, H.J. (1967). Prevalence and prognosis of individual clinical and pathogenic variables associated with colorectal carcinoma. *Cancer*, **20**, 1976.

SVENNEVIG, J.L., LUNDE, O.C., HOLTER, J. & BJORGSVIK, O. (1984). Lymphoid infiltration and prognosis in colorectal carcinoma. *Br. J. Cancer*, **49**, 375.

SYMONDS, D.A. & VICKERY, A.L. JR. (1976). Mucinous carcinoma of the colon and rectum. *Cancer*, **37**, 1891.

THOMPSON, J.J., HERLYN, M.F., ELDER, D.E., CLARK, W.H., STEPLEWSKI, Z. & KOPROWSKI, J. (1982). Expression of DR antigens in freshly human tumours. *Hybridoma*, **1**, 1616.

THORSBY, E., BERGHOLTZ, B., BERLE, E., BRAATHENS, L. & HIRSCHBERG, L. (1976). Involvement of HLA in T-cell immune responses. *Transplant Proc.*, **13**, 903.

UMPLEBY, H.C., HEINEMANN, D., SYMES, M.O. & WILLIAMSON, R.C.N. (1985). Expression of histocompatibility antigens and characterization of mononuclear cell infiltrates in normal and neoplastic colorectal tissues of humans. *J. Natl Cancer Inst.*, **74**, 1161.

VAN DEN INGH, H.F., GRIFFIOEN, G. & CORNELISSE, C.J. (1985). Flow cytometric detection of aneuploidy in colorectal adenomas. *Cancer Res.*, **45**, 3392.

WATT, A.G. & HOUSE, A.K. (1978). Colonic carcinoma. A quantitative assessment of lymphocyte infiltration at the periphery of colonic tumors related to prognosis. *Cancer*, **41**, 279.

WEISS, M.A., MICHAEL, J.G., PESCE, A.J. & DIPERSIO, L. (1981). Heterogeneity of $\beta_2$-microglobulin in human breast carcinoma. *Lab. Invest.*, **45**, 46.

WOLFMAN, E.F., ASTLER, V.B. & COLLIER, F.A. (1957). Mucoid adenocarcinomas of the colon and rectum. *Surgery*, **42**, 846.